# (*E*)-1-Methyl-4-styrylpyridinium iodide monohydrate

**DOI:** 10.1107/S1600536809040446

**Published:** 2009-10-10

**Authors:** Hoong-Kun Fun, Suchada Chantrapromma, Chanasuk Surasit, Kullapa Chanawanno

**Affiliations:** a X-ray Crystallography Unit, School of Physics, Universiti Sains Malaysia, 11800 USM, Penang, Malaysia; b Crystal Materials Research Unit, Department of Chemistry, Faculty of Science, Prince of Songkla University, Hat-Yai, Songkhla 90112, Thailand

## Abstract

In the title compound, C_14_H_14_N^+^·I^−^·H_2_O, the cation is essentially planar, with a dihedral angle of 2.55 (7)° between the pyridinium and phenyl rings, and exists in an *E* configuration with respect to the ethenyl bond. In the crystal structure, the cations are stacked in an anti­parallel manner along the *a* axis. The cation is linked to the water mol­ecule by a weak C—H⋯O inter­action, and the water mol­ecule is further linked to the I^−^ ion by O—H⋯I hydrogen bonds. The crystal structure is consolidated by these inter­actions and is further stabilized by a π–π inter­action between the pyridinium and phenyl rings with a centroid–centroid distance of 3.6850 (8) Å.

## Related literature

For bond-length data, see: Allen *et al.* (1987 ▶). For background to non-linear optical materials research, see: Chemla & Zyss (1987 ▶); Chia *et al.* (1995 ▶); Dittrich *et al.* (2003 ▶); Lin *et al.* (2002 ▶); Prasad & Williams (1991 ▶). For related structures, see: Chanawanno *et al.* (2008 ▶); Chantrapromma, Jindawong & Fun (2007 ▶); Chantrapromma, Jindawong, Fun & Patil (2007 ▶). For the stability of the temperature controller used in the data collection, see: Cosier & Glazer (1986 ▶).
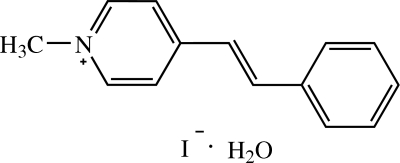


## Experimental

### 

#### Crystal data

C_14_H_14_N^+^·I^−^·H_2_O
*M* *_r_* = 341.18
Monoclinic, 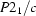

*a* = 7.3636 (1) Å
*b* = 10.5929 (1) Å
*c* = 18.2807 (2) Å
β = 106.770 (1)°
*V* = 1365.29 (3) Å^3^
*Z* = 4
Mo *K*α radiation
μ = 2.33 mm^−1^
*T* = 100 K
0.32 × 0.22 × 0.20 mm

#### Data collection

Bruker APEXII CCD area-detector diffractometer
Absorption correction: multi-scan (**SADABS**; Bruker, 2005 ▶) *T*_min_ = 0.524, *T*_max_ = 0.649
27548 measured reflections
6004 independent reflections
5307 reflections with *I* > 2σ(*I*)
*R*_int_ = 0.021

#### Refinement

*R*[*F* ^2^ > 2σ(*F* ^2^)] = 0.023
*wR*(*F* ^2^) = 0.058
*S* = 1.05
6004 reflections
163 parameters
H atoms treated by a mixture of independent and constrained refinement
Δρ_max_ = 1.32 e Å^−3^
Δρ_min_ = −0.56 e Å^−3^

### 

Data collection: *APEX2* (Bruker, 2005 ▶); cell refinement: *SAINT* (Bruker, 2005 ▶); data reduction: *SAINT*; program(s) used to solve structure: *SHELXTL* (Sheldrick, 2008 ▶); program(s) used to refine structure: *SHELXTL*; molecular graphics: *SHELXTL*; software used to prepare material for publication: *SHELXTL* and *PLATON* (Spek, 2009 ▶).

## Supplementary Material

Crystal structure: contains datablocks global, I. DOI: 10.1107/S1600536809040446/is2467sup1.cif

Structure factors: contains datablocks I. DOI: 10.1107/S1600536809040446/is2467Isup2.hkl

Additional supplementary materials:  crystallographic information; 3D view; checkCIF report

## Figures and Tables

**Table 1:** Hydrogen-bond geometry (Å, °)

*D*—H⋯*A*	*D*—H	H⋯*A*	*D*⋯*A*	*D*—H⋯*A*
O1*W*—H1*W*1⋯I1^i^	0.94 (3)	2.70 (3)	3.6458 (14)	177 (3)
O1*W*—H2*W*1⋯I1^ii^	0.93 (3)	2.66 (2)	3.5826 (12)	174 (2)
C14—H14*A*⋯O1*W*^ii^	0.96	2.52	3.3775 (19)	149

Symmetry codes: (i) 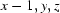; (ii) 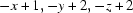.

**Fig. 1.**
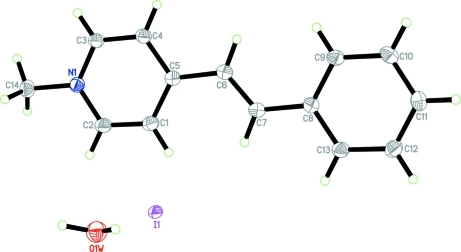
The molecular structure of the title compound, with 50% probability displacement ellipsoids and the atom-numbering scheme.

**Fig. 2.**
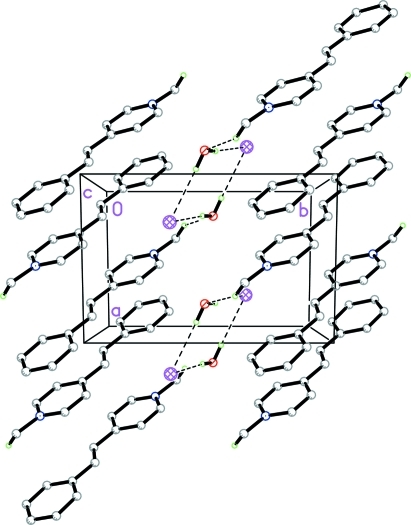
The crystal packing of the title compound viewed down the c axis. O—H···I hydrogen bonds and C—H···O interactions are shown as dashed lines.

## supplementary crystallographic information

### Comment

The design and synthesis of nonlinear optical (NLO) materials have been receiving much attention due to their numerous applications (Chemla & Zyss, 1987; Prasad & Williams, 1991). In the search for new organic NLO materials, aromatic compounds with extended π-conjugation system are extensively studied (Chia *et al.*, 1995; Dittrich *et al.*, 2003). Such materials require molecular hyperpolarizability and orientation in a noncentrosymmetric arrangement of the bulk material (Lin *et al.*, 2002; Prasad & Williams, 1991). During the course of our systematic studies of organic NLO materials, we have previously synthesized and reported the crystal structures of pyridinium and quinolinium iodide (Chanawanno *et al.*, 2008; Chantrapromma, Jindawong & Fun, 2007; Chantrapromma, Jindawong, Fun & Patil, 2007). Herein we report the crystal structure of the title pyridinium derivative (I). However (I) crystallizes in centrosymmetric *P*2_1_/c space group which precludes the second-order nonlinear optical properties.

The title compound consists of a C_14_H_14_N^+^ cation, an I^-^ anion and one water molecule (Fig. 1). The cation exists in an *E* configuration with respect to the C6═C7 ethenyl bond [1.3429 (18) Å] with the torsion angle of C5–C6–C7–C8 = -179.95 (13)°. The cation is essentially planar with the dihedral angles between the pyridinium [C1–C5/N1] and benzene rings being 2.55 (7)°. The ethenyl unit is co-planar with the pyridinium and benzene rings as indicated by the torsion angles C1–C5–C6–C7 = -1.4 (2)° and C6–C7–C8–C9 = 1.6 (2)°. The rms deviation from the plane through the cation is 0.027 (15) Å. The bond distances in the cation have normal values (Allen *et al.*, 1987) and comparable with the closely related compounds (Chanawanno *et al.*, 2008; Chantrapromma, Jindawong & Fun, 2007; Chantrapromma, Jindawong, Fun & Patil, 2007).

In the crystal packing (Fig. 2), the cations are stacked in an antiparallel manner along the *a* axis. The cation is linked with the water molecule by a C—H···O weak interaction. The water molecule is further linked with the I^-^ ion by O—H···I hydrogen bonds, forming a 3D network (Table 1). The crystal is consolidated by these interactions and further stabilized by π–π interactions with a distance of Cg_1_···Cg_2_^iii^ = 3.6850 (8) Å [symmetry code: (iii) -*x*, 1-*y*, 2-*z*]; Cg_1_ and Cg_2_ are the centroids of the C1–C5/N1 and C8–C13 rings, respectively.

### Experimental

(*E*)-1-Methyl-4-styrylpyridinium iodide was prepared by mixing 1:1:1 molar ratio solutions of 1,4-dimethylpyridinium iodide (2 g, 8.5 mmol), benzaldehyde (0.86 ml, 8.5 mmol) and piperidine (0.84 ml, 8.5 mmol) in methanol (40 ml). The resulting solution was refluxed for 3 h under a nitrogen atmosphere. The yellow solid which formed was filtered and washed with diethylether. Yellow block-shaped single crystals of the title compound suitable for *x*-ray structure determination were recrystallized from methanol by slow evaporation at room temperature over a few weeks (m.p. 489-490 K).

### Refinement

Water H atoms were located in a difference map and refined isotropically. The remaining H atoms were positioned geometrically and allowed to ride on their parent atoms, with *d*(C—H) = 0.93 Å for aromatic and CH and 0.96 Å for CH_3_ atoms. The *U*_iso_ values were constrained to be 1.5*U*_eq_ of the carrier atom for methyl H atoms and 1.2*U*_eq_ for the remaining H atoms. A rotating group model was used for the methyl groups. The highest residual electron density peak is located at 0.70 Å from I1 and the deepest hole is located at 0.54 Å from I1.

### Crystal data

**Table:**

C_14_H_14_N^+^·I^−^·H_2_O	*F*(000) = 672
*M**_r_* = 341.18	*D*_x_ = 1.660 Mg m^−^^3^
Monoclinic, *P*2_1_/*c*	Melting point = 489–490 K
Hall symbol: -P 2ybc	Mo *K*α radiation, λ = 0.71073 Å
*a* = 7.3636 (1) Å	Cell parameters from 6004 reflections
*b* = 10.5929 (1) Å	θ = 2.3–35.0°
*c* = 18.2807 (2) Å	µ = 2.33 mm^−^^1^
β = 106.770 (1)°	*T* = 100 K
*V* = 1365.29 (3) Å^3^	Block, yellow
*Z* = 4	0.32 × 0.22 × 0.20 mm

### Data collection

**Table:**

Bruker APEXII CCD area-detector diffractometer	6004 independent reflections
Radiation source: sealed tube	5307 reflections with *I* > 2σ(*I*)
graphite	*R*_int_ = 0.021
φ and ω scans	θ_max_ = 35.0°, θ_min_ = 2.3°
Absorption correction: multi-scan (*SADABS*; Bruker, 2005)	*h* = −11→11
*T*_min_ = 0.524, *T*_max_ = 0.649	*k* = −17→16
27548 measured reflections	*l* = −29→28

### Refinement

**Table:**

Refinement on *F*^2^	Primary atom site location: structure-invariant direct methods
Least-squares matrix: full	Secondary atom site location: difference Fourier map
*R*[*F*^2^ > 2σ(*F*^2^)] = 0.023	Hydrogen site location: inferred from neighbouring sites
*wR*(*F*^2^) = 0.058	H atoms treated by a mixture of independent and constrained refinement
*S* = 1.05	*w* = 1/[σ^2^(*F*_o_^2^) + (0.0248*P*)^2^ + 0.8184*P*] where *P* = (*F*_o_^2^ + 2*F*_c_^2^)/3
6004 reflections	(Δ/σ)_max_ = 0.004
163 parameters	Δρ_max_ = 1.32 e Å^−^^3^
0 restraints	Δρ_min_ = −0.56 e Å^−^^3^

### Special details

**Table:**

Experimental. The crystal was placed in the cold stream of an Oxford Cryosystems Cobra open-flow nitrogen cryostat (Cosier & Glazer, 1986) operating at 100.0 (1) K.
Geometry. All esds (except the esd in the dihedral angle between two l.s. planes) are estimated using the full covariance matrix. The cell esds are taken into account individually in the estimation of esds in distances, angles and torsion angles; correlations between esds in cell parameters are only used when they are defined by crystal symmetry. An approximate (isotropic) treatment of cell esds is used for estimating esds involving l.s. planes.
Refinement. Refinement of F^2^ against ALL reflections. The weighted R-factor wR and goodness of fit S are based on F^2^, conventional R-factors R are based on F, with F set to zero for negative F^2^. The threshold expression of F^2^ > 2sigma(F^2^) is used only for calculating R-factors(gt) etc. and is not relevant to the choice of reflections for refinement. R-factors based on F^2^ are statistically about twice as large as those based on F, and R- factors based on ALL data will be even larger.

### Fractional atomic coordinates and isotropic or equivalent isotropic displacement parameters (Å^2^)

**Table:**

	*x*	*y*	*z*	*U*_iso_*/*U*_eq_	
I1	0.742589 (15)	0.831113 (9)	0.885210 (5)	0.02548 (3)	
O1W	0.20711 (19)	0.98174 (12)	0.94686 (7)	0.0308 (2)	
H1W1	0.086 (4)	0.944 (3)	0.9327 (16)	0.060 (8)*	
H2W1	0.210 (4)	1.028 (2)	0.9901 (15)	0.048 (7)*	
N1	0.53960 (16)	0.74866 (11)	1.12888 (6)	0.01776 (19)	
C1	0.3622 (2)	0.68934 (13)	1.00380 (8)	0.0219 (2)	
H1A	0.3146	0.7096	0.9523	0.026*	
C2	0.4699 (2)	0.77624 (13)	1.05393 (8)	0.0218 (2)	
H2A	0.4948	0.8546	1.0360	0.026*	
C3	0.5064 (2)	0.63509 (12)	1.15607 (7)	0.0185 (2)	
H3A	0.5556	0.6174	1.2079	0.022*	
C4	0.4006 (2)	0.54563 (12)	1.10806 (7)	0.0187 (2)	
H4A	0.3796	0.4676	1.1275	0.022*	
C5	0.32393 (19)	0.57076 (12)	1.02979 (7)	0.0176 (2)	
C6	0.2111 (2)	0.47325 (12)	0.98062 (7)	0.0191 (2)	
H6A	0.1960	0.3967	1.0031	0.023*	
C7	0.12773 (19)	0.48617 (12)	0.90531 (7)	0.0188 (2)	
H7A	0.1434	0.5629	0.8832	0.023*	
C8	0.01415 (19)	0.38969 (12)	0.85514 (7)	0.0175 (2)	
C9	−0.0249 (2)	0.27106 (12)	0.88126 (8)	0.0195 (2)	
H9A	0.0259	0.2496	0.9324	0.023*	
C10	−0.1394 (2)	0.18542 (12)	0.83081 (9)	0.0214 (2)	
H10A	−0.1634	0.1065	0.8482	0.026*	
C11	−0.2182 (2)	0.21752 (13)	0.75423 (8)	0.0214 (2)	
H11A	−0.2975	0.1609	0.7210	0.026*	
C12	−0.1783 (2)	0.33396 (13)	0.72750 (8)	0.0226 (2)	
H12A	−0.2297	0.3551	0.6763	0.027*	
C13	−0.0616 (2)	0.41849 (13)	0.77750 (8)	0.0206 (2)	
H13A	−0.0331	0.4956	0.7592	0.025*	
C14	0.6473 (2)	0.84639 (13)	1.18135 (8)	0.0234 (3)	
H14A	0.7349	0.8866	1.1588	0.035*	
H14B	0.7160	0.8079	1.2288	0.035*	
H14C	0.5611	0.9082	1.1906	0.035*	

### Atomic displacement parameters (Å^2^)

**Table:**

	*U*^11^	*U*^22^	*U*^33^	*U*^12^	*U*^13^	*U*^23^
I1	0.03493 (6)	0.02076 (4)	0.01682 (4)	−0.00883 (3)	0.00120 (3)	0.00305 (3)
O1W	0.0324 (6)	0.0315 (6)	0.0294 (5)	0.0025 (5)	0.0103 (5)	−0.0004 (4)
N1	0.0178 (5)	0.0184 (4)	0.0172 (4)	−0.0012 (4)	0.0054 (4)	−0.0003 (3)
C1	0.0249 (7)	0.0220 (6)	0.0176 (5)	−0.0017 (5)	0.0043 (5)	0.0029 (4)
C2	0.0247 (7)	0.0209 (6)	0.0199 (5)	−0.0022 (5)	0.0065 (5)	0.0038 (4)
C3	0.0203 (6)	0.0177 (5)	0.0183 (5)	0.0011 (4)	0.0068 (4)	0.0015 (4)
C4	0.0207 (6)	0.0174 (5)	0.0191 (5)	0.0005 (4)	0.0074 (4)	0.0018 (4)
C5	0.0166 (5)	0.0181 (5)	0.0182 (5)	0.0008 (4)	0.0055 (4)	0.0011 (4)
C6	0.0202 (6)	0.0177 (5)	0.0194 (5)	0.0002 (4)	0.0060 (4)	0.0017 (4)
C7	0.0193 (6)	0.0181 (5)	0.0192 (5)	0.0008 (4)	0.0058 (4)	0.0025 (4)
C8	0.0159 (5)	0.0178 (5)	0.0188 (5)	0.0006 (4)	0.0051 (4)	0.0005 (4)
C9	0.0186 (6)	0.0181 (5)	0.0212 (5)	0.0024 (4)	0.0049 (4)	0.0033 (4)
C10	0.0207 (6)	0.0158 (5)	0.0281 (6)	0.0012 (4)	0.0077 (5)	0.0023 (4)
C11	0.0198 (6)	0.0208 (6)	0.0238 (6)	−0.0012 (5)	0.0068 (5)	−0.0047 (4)
C12	0.0243 (7)	0.0251 (6)	0.0178 (5)	−0.0005 (5)	0.0054 (5)	0.0005 (4)
C13	0.0212 (6)	0.0207 (5)	0.0200 (5)	−0.0015 (5)	0.0064 (5)	0.0026 (4)
C14	0.0248 (7)	0.0228 (6)	0.0224 (6)	−0.0050 (5)	0.0065 (5)	−0.0044 (4)

### Geometric parameters (Å, °)

**Table:**

O1W—H1W1	0.94 (3)	C7—C8	1.4637 (18)
O1W—H2W1	0.93 (3)	C7—H7A	0.9300
N1—C2	1.3491 (17)	C8—C13	1.4005 (18)
N1—C3	1.3507 (17)	C8—C9	1.4032 (19)
N1—C14	1.4772 (18)	C9—C10	1.391 (2)
C1—C2	1.377 (2)	C9—H9A	0.9300
C1—C5	1.4003 (19)	C10—C11	1.394 (2)
C1—H1A	0.9300	C10—H10A	0.9300
C2—H2A	0.9300	C11—C12	1.389 (2)
C3—C4	1.3711 (19)	C11—H11A	0.9300
C3—H3A	0.9300	C12—C13	1.387 (2)
C4—C5	1.4039 (18)	C12—H12A	0.9300
C4—H4A	0.9300	C13—H13A	0.9300
C5—C6	1.4608 (19)	C14—H14A	0.9600
C6—C7	1.3429 (18)	C14—H14B	0.9600
C6—H6A	0.9300	C14—H14C	0.9600
			
H1W1—O1W—H2W1	104 (2)	C13—C8—C9	118.63 (12)
C2—N1—C3	120.69 (12)	C13—C8—C7	118.12 (12)
C2—N1—C14	118.90 (12)	C9—C8—C7	123.24 (11)
C3—N1—C14	120.37 (11)	C10—C9—C8	120.19 (12)
C2—C1—C5	120.49 (12)	C10—C9—H9A	119.9
C2—C1—H1A	119.8	C8—C9—H9A	119.9
C5—C1—H1A	119.8	C9—C10—C11	120.24 (12)
N1—C2—C1	120.55 (12)	C9—C10—H10A	119.9
N1—C2—H2A	119.7	C11—C10—H10A	119.9
C1—C2—H2A	119.7	C12—C11—C10	120.06 (13)
N1—C3—C4	120.65 (12)	C12—C11—H11A	120.0
N1—C3—H3A	119.7	C10—C11—H11A	120.0
C4—C3—H3A	119.7	C13—C12—C11	119.69 (13)
C3—C4—C5	120.56 (12)	C13—C12—H12A	120.2
C3—C4—H4A	119.7	C11—C12—H12A	120.2
C5—C4—H4A	119.7	C12—C13—C8	121.14 (12)
C1—C5—C4	117.05 (12)	C12—C13—H13A	119.4
C1—C5—C6	124.05 (12)	C8—C13—H13A	119.4
C4—C5—C6	118.90 (12)	N1—C14—H14A	109.5
C7—C6—C5	124.71 (12)	N1—C14—H14B	109.5
C7—C6—H6A	117.6	H14A—C14—H14B	109.5
C5—C6—H6A	117.6	N1—C14—H14C	109.5
C6—C7—C8	125.51 (12)	H14A—C14—H14C	109.5
C6—C7—H7A	117.2	H14B—C14—H14C	109.5
C8—C7—H7A	117.2		
			
C3—N1—C2—C1	0.5 (2)	C5—C6—C7—C8	−179.95 (13)
C14—N1—C2—C1	−177.34 (14)	C6—C7—C8—C13	−179.47 (14)
C5—C1—C2—N1	−0.2 (2)	C6—C7—C8—C9	1.6 (2)
C2—N1—C3—C4	−0.2 (2)	C13—C8—C9—C10	−1.1 (2)
C14—N1—C3—C4	177.64 (13)	C7—C8—C9—C10	177.78 (13)
N1—C3—C4—C5	−0.4 (2)	C8—C9—C10—C11	−0.9 (2)
C2—C1—C5—C4	−0.3 (2)	C9—C10—C11—C12	1.8 (2)
C2—C1—C5—C6	179.97 (14)	C10—C11—C12—C13	−0.7 (2)
C3—C4—C5—C1	0.7 (2)	C11—C12—C13—C8	−1.3 (2)
C3—C4—C5—C6	−179.63 (13)	C9—C8—C13—C12	2.2 (2)
C1—C5—C6—C7	−1.4 (2)	C7—C8—C13—C12	−176.72 (13)
C4—C5—C6—C7	178.88 (14)		

### Hydrogen-bond geometry (Å, °)

**Table:**

*D*—H···*A*	*D*—H	H···*A*	*D*···*A*	*D*—H···*A*
O1W—H1W1···I1^i^	0.94 (3)	2.70 (3)	3.6458 (14)	177 (3)
O1W—H2W1···I1^ii^	0.93 (3)	2.66 (2)	3.5826 (12)	174 (2)
C14—H14A···O1W^ii^	0.96	2.52	3.3775 (19)	149

Symmetry codes: (i) *x*−1, *y*, *z*; (ii) −*x*+1, −*y*+2, −*z*+2.
